# Crystal structure of methyl *N*-ferro­cenyl­carbamate

**DOI:** 10.1107/S2056989015000043

**Published:** 2015-01-17

**Authors:** Alan J. Rodríguez, J. Martin. E. Quirke, Aida O. Diouf

**Affiliations:** a Department of Chemistry and Biochemistry, Florida International University, 11200 SW 8th Street, Miami, Florida 33199, USA; b Department of Chemistry and Biochemistry, Albright College, 1621 North 13th Street, Reading, PA 19604, USA

**Keywords:** crystal structure, ferrocene, carbamate, ferrocenoyl azide derivatives, *N*-ferrocenoyl­methyl­carbamate

## Abstract

The asymmetric unit of the title compound, [Fe(C_5_H_5_)(C_7_H_8_NO_2_)], contains two independent mol­ecules consisting of a ferrocenyl moiety and a nitro­gen-bound methyl carbamate. These units are almost perpendicular to each other, making dihedral angles of 87.74 (9) and 87.32 (8)°. In each independent mol­ecule, the cyclo­penta­dienyl rings deviate slightly from an eclipsed conformation and lie virtually parallel [dihedral angles = 1.42 (15) and 0.49 (13)°]. In the crystal, mol­ecules are linked by N—H⋯O hydrogen bonds into chains along the *a*-axis direction.

## Related literature   

For the synthesis and fragmentation mechanism of the title compound, see: Van Berkel *et al.* (1998 ▸); Quirke *et al.* (2001 ▸). For related ferrocenyl derivatives, see: Barišić *et al.* (2011 ▸).
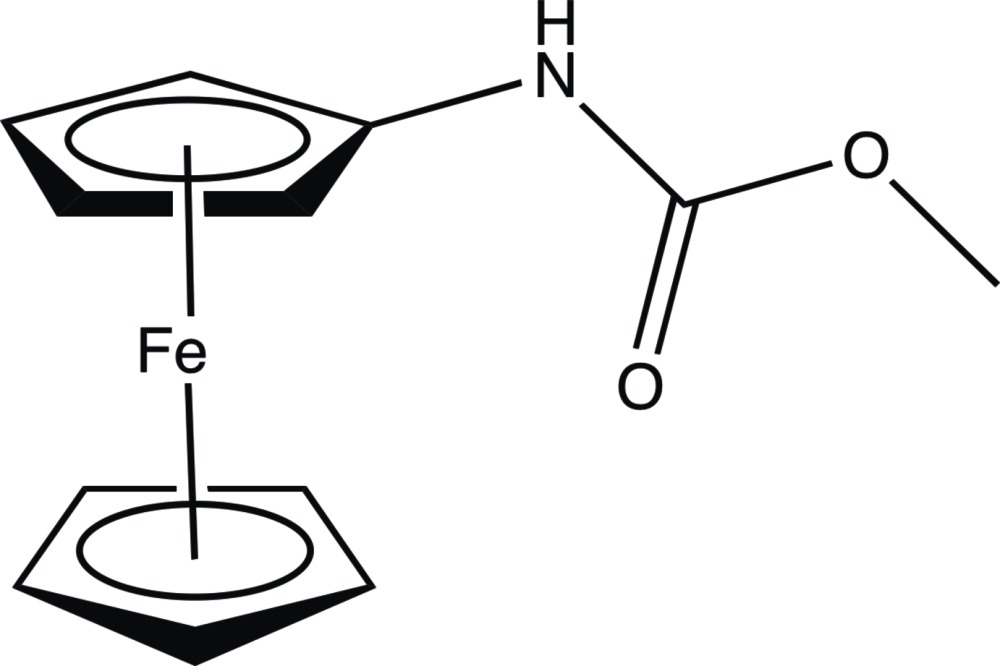


## Experimental   

### Crystal data  

[Fe(C_5_H_5_)(C_7_H_8_NO_2_)]
*M* *_r_* = 259.08
Triclinic, 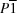

*a* = 10.1224 (5) Å
*b* = 10.7849 (5) Å
*c* = 11.0445 (5) Å
α = 76.156 (13)°
β = 73.960 (13)°
γ = 89.059 (14)°
*V* = 1123.52 (9) Å^3^
*Z* = 4
Mo *K*α radiation
μ = 1.33 mm^−1^
*T* = 296 K
0.38 × 0.20 × 0.15 mm

### Data collection  

Bruker D8 Quest diffractometer
Absorption correction: multi-scan (*SADABS*; Bruker, 2013 ▸) *T*_min_ = 0.75, *T*_max_ = 0.83
24698 measured reflections
5586 independent reflections
4560 reflections with *I* > 2σ(*I*)
*R*_int_ = 0.022

### Refinement  

*R*[*F* ^2^ > 2σ(*F* ^2^)] = 0.030
*wR*(*F* ^2^) = 0.071
*S* = 0.99
5586 reflections
299 parameters
H atoms treated by a mixture of independent and constrained refinement
Δρ_max_ = 0.33 e Å^−3^
Δρ_min_ = −0.26 e Å^−3^

### 

Data collection: *APEX2* (Bruker, 2014 ▸); cell refinement: *SAINT* (Bruker, 2013 ▸); data reduction: *SAINT*; program(s) used to solve structure: *SHELXT-2014* (Sheldrick, 2015 ▸); program(s) used to refine structure: *SHELXL2014*/6 (Sheldrick, 2008 ▸); molecular graphics: *Mercury* (Macrae *et al.*, 2006 ▸); software used to prepare material for publication: *publCIF* (Westrip, 2010 ▸).

## Supplementary Material

Crystal structure: contains datablock(s) I, New_Global_Publ_Block. DOI: 10.1107/S2056989015000043/pj2017sup1.cif

Structure factors: contains datablock(s) I. DOI: 10.1107/S2056989015000043/pj2017Isup2.hkl

Supporting information file. DOI: 10.1107/S2056989015000043/pj2017Isup3.cdx

**Fig. 1.**
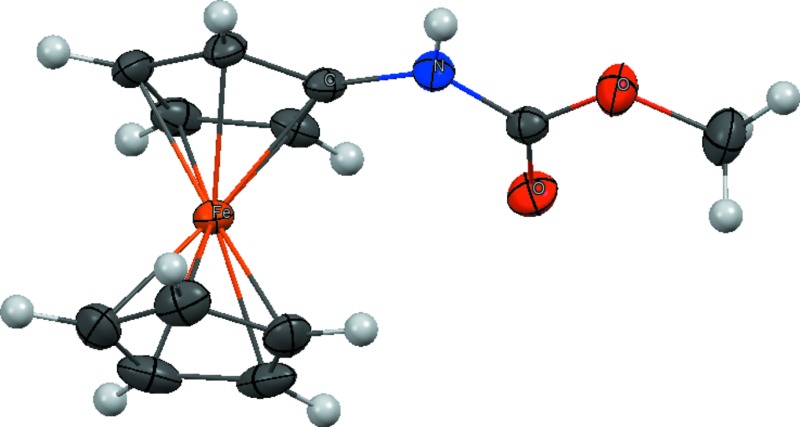
The molecular structure of the title compound with atoms drawn as ellipsoids at the 30% probability level.

. DOI: 10.1107/S2056989015000043/pj2017fig1.tif
The mol­ecular structure of the title compound with atoms drawn as ellipsoids at the 30% probability level.

**Fig. 2.**
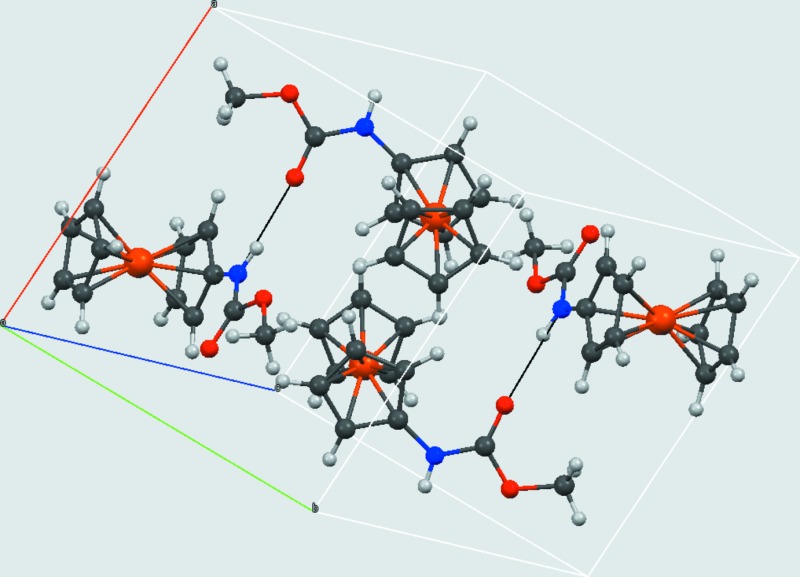
Packing diagram of the title compound showing intermolecular H-bonding interactions.

. DOI: 10.1107/S2056989015000043/pj2017fig2.tif
Packing diagram of the title compound showing inter­molecular H-bonding inter­actions.

CCDC reference: 1041904

Additional supporting information:  crystallographic information; 3D view; checkCIF report

## Figures and Tables

**Table 1:** Hydrogen-bond geometry (, )

*D*H*A*	*D*H	H*A*	*D* *A*	*D*H*A*
N1H1O3^i^	0.82(2)	2.18(2)	2.971(2)	162.5(19)
N2H2O1	0.80(2)	2.18(2)	2.9605(19)	166(2)

Symmetry code: (i) 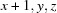.

## supplementary crystallographic information

### Crystal data

**Table:**

[Fe(C_5_H_5_)(C_7_H_8_NO_2_)]	*Z* = 4
*M**_r_* = 259.08	*F*(000) = 536
Triclinic, *P*1	*D*_x_ = 1.532 Mg m^−^^3^
*a* = 10.1224 (5) Å	Mo *K*α radiation, λ = 0.71073 Å
*b* = 10.7849 (5) Å	Cell parameters from 120 reflections
*c* = 11.0445 (5) Å	θ = 2.9–22.8°
α = 76.156 (13)°	µ = 1.33 mm^−^^1^
β = 73.960 (13)°	*T* = 296 K
γ = 89.059 (14)°	Needle, lusterous yellow
*V* = 1123.52 (9) Å^3^	0.38 × 0.20 × 0.15 mm

### Data collection

**Table:**

Bruker D8 Quest diffractometer	5586 independent reflections
Radiation source: fine-focus tube	4560 reflections with *I* > 2σ(*I*)
Detector resolution: 10.4167 pixels mm^-1^	*R*_int_ = 0.022
ω scans	θ_max_ = 28.3°, θ_min_ = 2.9°
Absorption correction: multi-scan (*SADABS*; Bruker, 2013)	*h* = −13→13
*T*_min_ = 0.75, *T*_max_ = 0.83	*k* = −14→14
24698 measured reflections	*l* = −14→14

### Refinement

**Table:**

Refinement on *F*^2^	0 restraints
Least-squares matrix: full	Hydrogen site location: mixed
*R*[*F*^2^ > 2σ(*F*^2^)] = 0.030	H atoms treated by a mixture of independent and constrained refinement
*wR*(*F*^2^) = 0.071	*w* = 1/[σ^2^(*F*_o_^2^) + (0.0293*P*)^2^ + 0.5563*P*] where *P* = (*F*_o_^2^ + 2*F*_c_^2^)/3
*S* = 0.99	(Δ/σ)_max_ = 0.001
5586 reflections	Δρ_max_ = 0.33 e Å^−^^3^
299 parameters	Δρ_min_ = −0.26 e Å^−^^3^

### Special details

**Table:**

Geometry. All e.s.d.'s (except the e.s.d. in the dihedral angle between two l.s. planes) are estimated using the full covariance matrix. The cell e.s.d.'s are taken into account individually in the estimation of e.s.d.'s in distances, angles and torsion angles; correlations between e.s.d.'s in cell parameters are only used when they are defined by crystal symmetry. An approximate (isotropic) treatment of cell e.s.d.'s is used for estimating e.s.d.'s involving l.s. planes.

### Fractional atomic coordinates and isotropic or equivalent isotropic displacement parameters (Å^2^)

**Table:**

	*x*	*y*	*z*	*U*_iso_*/*U*_eq_	
Fe1	0.75463 (2)	0.60308 (2)	0.31717 (2)	0.03709 (7)	
Fe2	0.25564 (2)	−0.00924 (2)	0.31751 (2)	0.03430 (7)	
C1	0.82395 (18)	0.42097 (17)	0.34739 (17)	0.0389 (4)	
C2	0.6871 (2)	0.42219 (18)	0.42686 (18)	0.0453 (4)	
H2A	0.6064	0.3734	0.4264	0.054*	
C3	0.6888 (2)	0.5061 (2)	0.50739 (19)	0.0546 (5)	
H3	0.6091	0.5257	0.5726	0.065*	
C4	0.8241 (2)	0.5576 (2)	0.4772 (2)	0.0560 (5)	
H4	0.8548	0.6186	0.5181	0.067*	
C5	0.9086 (2)	0.5057 (2)	0.3776 (2)	0.0485 (5)	
H5	1.0077	0.5243	0.3376	0.058*	
C6	0.6410 (3)	0.6599 (2)	0.1906 (3)	0.0737 (8)	
H6	0.5717	0.6071	0.1761	0.088*	
C7	0.6172 (3)	0.7354 (3)	0.2817 (3)	0.0780 (8)	
H7	0.5283	0.7447	0.3419	0.094*	
C8	0.7425 (3)	0.7959 (2)	0.2696 (3)	0.0750 (8)	
H8	0.7574	0.8549	0.3202	0.090*	
C9	0.8412 (3)	0.7591 (2)	0.1727 (2)	0.0691 (7)	
H9	0.9390	0.7868	0.1439	0.083*	
C10	0.7808 (3)	0.6751 (2)	0.1237 (2)	0.0684 (7)	
H10	0.8276	0.6346	0.0541	0.082*	
C13	0.32117 (17)	0.15345 (17)	0.35388 (16)	0.0372 (4)	
C14	0.18305 (18)	0.11686 (18)	0.42921 (17)	0.0425 (4)	
H14	0.1028	0.1685	0.4272	0.051*	
C15	0.1830 (2)	−0.0073 (2)	0.50823 (18)	0.0505 (5)	
H15	0.1021	−0.0572	0.5706	0.061*	
C16	0.3191 (2)	−0.0481 (2)	0.48139 (18)	0.0502 (5)	
H16	0.3490	−0.1306	0.5222	0.060*	
C17	0.40510 (19)	0.05143 (18)	0.38491 (18)	0.0438 (4)	
H17	0.5047	0.0500	0.3472	0.053*	
C18	0.2790 (3)	0.0257 (2)	0.12343 (19)	0.0569 (5)	
H18	0.3187	0.1045	0.0583	0.068*	
C19	0.1395 (2)	−0.0010 (3)	0.1929 (2)	0.0633 (7)	
H19	0.0644	0.0561	0.1849	0.076*	
C20	0.1266 (2)	−0.1246 (3)	0.2754 (2)	0.0645 (6)	
H20	0.0410	−0.1687	0.3352	0.077*	
C21	0.2574 (3)	−0.1733 (2)	0.2569 (2)	0.0614 (6)	
H21	0.2796	−0.2575	0.3017	0.074*	
C22	0.3509 (2)	−0.0813 (2)	0.1635 (2)	0.0572 (5)	
H22	0.4502	−0.0900	0.1316	0.069*	
C11	0.79773 (17)	0.27959 (16)	0.21235 (16)	0.0371 (4)	
C12	0.8079 (3)	0.1308 (2)	0.0852 (2)	0.0628 (6)	
H12A	0.7741	0.1816	0.0171	0.094*	
H12B	0.7322	0.0844	0.1536	0.094*	
H12C	0.8707	0.0716	0.0512	0.094*	
C23	0.29892 (18)	0.35980 (17)	0.21196 (17)	0.0384 (4)	
C24	0.3153 (3)	0.5670 (2)	0.0756 (2)	0.0686 (6)	
H24A	0.3801	0.6390	0.0357	0.103*	
H24B	0.2388	0.5897	0.1386	0.103*	
H24C	0.2831	0.5424	0.0103	0.103*	
N1	0.87534 (16)	0.34529 (15)	0.25925 (16)	0.0418 (3)	
N2	0.37378 (16)	0.27173 (15)	0.26751 (16)	0.0414 (3)	
O1	0.67396 (13)	0.27931 (14)	0.23547 (14)	0.0507 (3)	
O2	0.87800 (14)	0.21268 (14)	0.13527 (14)	0.0526 (3)	
O3	0.17679 (13)	0.35027 (14)	0.22624 (15)	0.0551 (4)	
O4	0.38073 (14)	0.46244 (13)	0.13837 (14)	0.0556 (4)	
H2	0.455 (2)	0.284 (2)	0.248 (2)	0.049 (6)*	
H1	0.959 (2)	0.3413 (19)	0.2356 (19)	0.044 (6)*	

### Atomic displacement parameters (Å^2^)

**Table:**

	*U*^11^	*U*^22^	*U*^33^	*U*^12^	*U*^13^	*U*^23^
Fe1	0.03477 (14)	0.03807 (14)	0.04083 (14)	0.00500 (10)	−0.01523 (11)	−0.00900 (10)
Fe2	0.03208 (13)	0.04001 (14)	0.03383 (13)	0.00118 (10)	−0.01287 (10)	−0.01047 (10)
C1	0.0358 (9)	0.0422 (9)	0.0416 (9)	0.0111 (7)	−0.0151 (7)	−0.0113 (7)
C2	0.0422 (10)	0.0412 (10)	0.0439 (10)	0.0078 (8)	−0.0052 (8)	−0.0026 (8)
C3	0.0632 (13)	0.0574 (12)	0.0382 (10)	0.0231 (10)	−0.0092 (9)	−0.0096 (9)
C4	0.0704 (14)	0.0631 (13)	0.0521 (12)	0.0266 (11)	−0.0359 (11)	−0.0270 (10)
C5	0.0429 (10)	0.0597 (12)	0.0564 (12)	0.0164 (9)	−0.0290 (9)	−0.0234 (10)
C6	0.0788 (17)	0.0598 (14)	0.0917 (19)	−0.0075 (12)	−0.0635 (16)	0.0112 (13)
C7	0.0659 (16)	0.0719 (17)	0.0765 (17)	0.0334 (13)	−0.0146 (13)	0.0105 (14)
C8	0.122 (2)	0.0374 (11)	0.0737 (17)	0.0061 (13)	−0.0441 (17)	−0.0096 (11)
C9	0.0667 (15)	0.0652 (15)	0.0657 (15)	−0.0199 (12)	−0.0221 (12)	0.0082 (12)
C10	0.096 (2)	0.0683 (15)	0.0440 (12)	0.0130 (13)	−0.0288 (12)	−0.0086 (11)
C13	0.0331 (8)	0.0445 (9)	0.0362 (9)	−0.0047 (7)	−0.0128 (7)	−0.0103 (7)
C14	0.0364 (9)	0.0516 (11)	0.0406 (9)	−0.0068 (8)	−0.0043 (7)	−0.0205 (8)
C15	0.0548 (12)	0.0611 (12)	0.0327 (9)	−0.0195 (10)	−0.0064 (8)	−0.0114 (8)
C16	0.0630 (13)	0.0494 (11)	0.0414 (10)	−0.0100 (9)	−0.0291 (9)	0.0004 (8)
C17	0.0376 (9)	0.0510 (11)	0.0462 (10)	−0.0033 (8)	−0.0229 (8)	−0.0050 (8)
C18	0.0816 (16)	0.0578 (13)	0.0376 (10)	0.0065 (11)	−0.0251 (10)	−0.0140 (9)
C19	0.0644 (14)	0.0859 (17)	0.0729 (15)	0.0335 (12)	−0.0479 (12)	−0.0507 (14)
C20	0.0566 (13)	0.0851 (17)	0.0631 (14)	−0.0173 (12)	−0.0159 (11)	−0.0393 (13)
C21	0.0897 (17)	0.0461 (11)	0.0613 (13)	0.0114 (11)	−0.0329 (13)	−0.0242 (10)
C22	0.0531 (12)	0.0751 (15)	0.0506 (12)	0.0185 (11)	−0.0139 (10)	−0.0304 (11)
C11	0.0352 (9)	0.0367 (9)	0.0373 (9)	0.0032 (7)	−0.0123 (7)	−0.0029 (7)
C12	0.0772 (16)	0.0549 (13)	0.0674 (14)	0.0007 (11)	−0.0290 (12)	−0.0256 (11)
C23	0.0358 (9)	0.0428 (9)	0.0402 (9)	0.0051 (7)	−0.0126 (7)	−0.0148 (7)
C24	0.0804 (17)	0.0506 (13)	0.0726 (15)	0.0156 (12)	−0.0287 (13)	−0.0035 (11)
N1	0.0265 (7)	0.0508 (9)	0.0540 (9)	0.0074 (6)	−0.0129 (7)	−0.0226 (7)
N2	0.0256 (7)	0.0432 (8)	0.0529 (9)	−0.0026 (6)	−0.0125 (7)	−0.0048 (7)
O1	0.0323 (7)	0.0616 (9)	0.0608 (8)	0.0011 (6)	−0.0182 (6)	−0.0139 (7)
O2	0.0460 (8)	0.0602 (9)	0.0608 (9)	0.0062 (6)	−0.0160 (7)	−0.0313 (7)
O3	0.0335 (7)	0.0634 (9)	0.0714 (10)	0.0089 (6)	−0.0211 (7)	−0.0153 (7)
O4	0.0485 (8)	0.0472 (8)	0.0623 (9)	0.0024 (6)	−0.0161 (7)	0.0039 (7)

### Geometric parameters (Å, º)

**Table:**

Fe1—C7	2.023 (2)	C9—H9	0.9800
Fe1—C6	2.027 (2)	C10—H10	0.9800
Fe1—C4	2.0296 (19)	C13—N2	1.407 (2)
Fe1—C8	2.030 (2)	C13—C17	1.414 (3)
Fe1—C9	2.034 (2)	C13—C14	1.422 (2)
Fe1—C10	2.035 (2)	C14—C15	1.413 (3)
Fe1—C3	2.037 (2)	C14—H14	0.9800
Fe1—C5	2.0410 (18)	C15—C16	1.413 (3)
Fe1—C2	2.0484 (19)	C15—H15	0.9800
Fe1—C1	2.0571 (17)	C16—C17	1.420 (3)
Fe2—C19	2.0292 (19)	C16—H16	0.9800
Fe2—C18	2.0319 (19)	C17—H17	0.9800
Fe2—C22	2.032 (2)	C18—C22	1.401 (3)
Fe2—C16	2.0336 (18)	C18—C19	1.405 (3)
Fe2—C20	2.034 (2)	C18—H18	0.9800
Fe2—C21	2.034 (2)	C19—C20	1.409 (4)
Fe2—C15	2.0352 (18)	C19—H19	0.9800
Fe2—C17	2.0394 (17)	C20—C21	1.396 (3)
Fe2—C14	2.0447 (18)	C20—H20	0.9800
Fe2—C13	2.0500 (17)	C21—C22	1.395 (3)
C1—N1	1.404 (2)	C21—H21	0.9800
C1—C5	1.418 (3)	C22—H22	0.9800
C1—C2	1.422 (2)	C11—O1	1.207 (2)
C2—C3	1.416 (3)	C11—O2	1.341 (2)
C2—H2A	0.9800	C11—N1	1.344 (2)
C3—C4	1.407 (3)	C12—O2	1.435 (2)
C3—H3	0.9800	C12—H12A	0.9600
C4—C5	1.420 (3)	C12—H12B	0.9600
C4—H4	0.9800	C12—H12C	0.9600
C5—H5	0.9800	C23—O3	1.205 (2)
C6—C10	1.395 (4)	C23—O4	1.341 (2)
C6—C7	1.409 (4)	C23—N2	1.344 (2)
C6—H6	0.9800	C24—O4	1.430 (3)
C7—C8	1.393 (4)	C24—H24A	0.9600
C7—H7	0.9800	C24—H24B	0.9600
C8—C9	1.379 (4)	C24—H24C	0.9600
C8—H8	0.9800	N1—H1	0.82 (2)
C9—C10	1.386 (3)	N2—H2	0.80 (2)
			
C7—Fe1—C6	40.72 (12)	C4—C5—H5	126.2
C7—Fe1—C4	126.60 (11)	Fe1—C5—H5	126.2
C6—Fe1—C4	165.02 (12)	C10—C6—C7	107.6 (2)
C7—Fe1—C8	40.21 (12)	C10—C6—Fe1	70.21 (13)
C6—Fe1—C8	67.83 (11)	C7—C6—Fe1	69.50 (13)
C4—Fe1—C8	107.38 (10)	C10—C6—H6	126.2
C7—Fe1—C9	67.02 (11)	C7—C6—H6	126.2
C6—Fe1—C9	67.10 (10)	Fe1—C6—H6	126.2
C4—Fe1—C9	118.98 (10)	C8—C7—C6	107.7 (2)
C8—Fe1—C9	39.67 (11)	C8—C7—Fe1	70.14 (14)
C7—Fe1—C10	67.77 (11)	C6—C7—Fe1	69.79 (13)
C6—Fe1—C10	40.17 (11)	C8—C7—H7	126.1
C4—Fe1—C10	152.75 (11)	C6—C7—H7	126.1
C8—Fe1—C10	67.40 (11)	Fe1—C7—H7	126.1
C9—Fe1—C10	39.84 (10)	C9—C8—C7	107.8 (2)
C7—Fe1—C3	108.40 (10)	C9—C8—Fe1	70.32 (14)
C6—Fe1—C3	128.18 (11)	C7—C8—Fe1	69.64 (13)
C4—Fe1—C3	40.48 (9)	C9—C8—H8	126.1
C8—Fe1—C3	119.16 (10)	C7—C8—H8	126.1
C9—Fe1—C3	152.71 (10)	Fe1—C8—H8	126.1
C10—Fe1—C3	165.88 (11)	C8—C9—C10	109.3 (2)
C7—Fe1—C5	163.91 (12)	C8—C9—Fe1	70.01 (14)
C6—Fe1—C5	153.42 (11)	C10—C9—Fe1	70.12 (13)
C4—Fe1—C5	40.82 (8)	C8—C9—H9	125.4
C8—Fe1—C5	126.38 (11)	C10—C9—H9	125.4
C9—Fe1—C5	108.18 (10)	Fe1—C9—H9	125.4
C10—Fe1—C5	119.19 (10)	C9—C10—C6	107.6 (2)
C3—Fe1—C5	68.34 (9)	C9—C10—Fe1	70.04 (13)
C7—Fe1—C2	120.04 (10)	C6—C10—Fe1	69.62 (13)
C6—Fe1—C2	109.18 (9)	C9—C10—H10	126.2
C4—Fe1—C2	68.36 (9)	C6—C10—H10	126.2
C8—Fe1—C2	153.54 (11)	Fe1—C10—H10	126.2
C9—Fe1—C2	165.55 (10)	N2—C13—C17	123.13 (16)
C10—Fe1—C2	128.38 (10)	N2—C13—C14	128.28 (17)
C3—Fe1—C2	40.57 (8)	C17—C13—C14	108.49 (16)
C5—Fe1—C2	68.48 (8)	N2—C13—Fe2	129.73 (12)
C7—Fe1—C1	154.45 (11)	C17—C13—Fe2	69.37 (10)
C6—Fe1—C1	120.32 (10)	C14—C13—Fe2	69.48 (10)
C4—Fe1—C1	68.13 (8)	C15—C14—C13	107.36 (17)
C8—Fe1—C1	164.23 (11)	C15—C14—Fe2	69.38 (11)
C9—Fe1—C1	128.00 (10)	C13—C14—Fe2	69.89 (10)
C10—Fe1—C1	109.21 (9)	C15—C14—H14	126.3
C3—Fe1—C1	67.97 (8)	C13—C14—H14	126.3
C5—Fe1—C1	40.48 (7)	Fe2—C14—H14	126.3
C2—Fe1—C1	40.54 (7)	C16—C15—C14	108.53 (17)
C19—Fe2—C18	40.49 (10)	C16—C15—Fe2	69.62 (11)
C19—Fe2—C22	67.75 (9)	C14—C15—Fe2	70.10 (10)
C18—Fe2—C22	40.34 (9)	C16—C15—H15	125.7
C19—Fe2—C16	162.31 (10)	C14—C15—H15	125.7
C18—Fe2—C16	155.74 (9)	Fe2—C15—H15	125.7
C22—Fe2—C16	121.11 (9)	C15—C16—C17	108.03 (18)
C19—Fe2—C20	40.57 (10)	C15—C16—Fe2	69.75 (11)
C18—Fe2—C20	68.05 (10)	C17—C16—Fe2	69.82 (10)
C22—Fe2—C20	67.53 (9)	C15—C16—H16	126.0
C16—Fe2—C20	125.34 (10)	C17—C16—H16	126.0
C19—Fe2—C21	67.83 (9)	Fe2—C16—H16	126.0
C18—Fe2—C21	67.84 (9)	C13—C17—C16	107.58 (17)
C22—Fe2—C21	40.11 (10)	C13—C17—Fe2	70.18 (9)
C16—Fe2—C21	108.15 (9)	C16—C17—Fe2	69.38 (10)
C20—Fe2—C21	40.13 (10)	C13—C17—H17	126.2
C19—Fe2—C15	125.56 (9)	C16—C17—H17	126.2
C18—Fe2—C15	162.01 (9)	Fe2—C17—H17	126.2
C22—Fe2—C15	156.46 (9)	C22—C18—C19	107.5 (2)
C16—Fe2—C15	40.63 (9)	C22—C18—Fe2	69.84 (12)
C20—Fe2—C15	108.67 (9)	C19—C18—Fe2	69.65 (12)
C21—Fe2—C15	121.89 (9)	C22—C18—H18	126.2
C19—Fe2—C17	155.73 (10)	C19—C18—H18	126.2
C18—Fe2—C17	120.36 (9)	Fe2—C18—H18	126.2
C22—Fe2—C17	107.46 (9)	C18—C19—C20	107.9 (2)
C16—Fe2—C17	40.80 (7)	C18—C19—Fe2	69.86 (11)
C20—Fe2—C17	161.74 (10)	C20—C19—Fe2	69.90 (12)
C21—Fe2—C17	124.84 (9)	C18—C19—H19	126.1
C15—Fe2—C17	68.45 (8)	C20—C19—H19	126.1
C19—Fe2—C14	108.02 (8)	Fe2—C19—H19	126.1
C18—Fe2—C14	124.87 (9)	C21—C20—C19	107.9 (2)
C22—Fe2—C14	161.60 (9)	C21—C20—Fe2	69.95 (12)
C16—Fe2—C14	68.44 (9)	C19—C20—Fe2	69.53 (12)
C20—Fe2—C14	121.77 (9)	C21—C20—H20	126.1
C21—Fe2—C14	156.75 (9)	C19—C20—H20	126.1
C15—Fe2—C14	40.53 (8)	Fe2—C20—H20	126.1
C17—Fe2—C14	68.59 (8)	C22—C21—C20	108.2 (2)
C19—Fe2—C13	121.31 (9)	C22—C21—Fe2	69.85 (12)
C18—Fe2—C13	107.63 (8)	C20—C21—Fe2	69.92 (12)
C22—Fe2—C13	124.88 (9)	C22—C21—H21	125.9
C16—Fe2—C13	68.09 (7)	C20—C21—H21	125.9
C20—Fe2—C13	156.81 (9)	Fe2—C21—H21	125.9
C21—Fe2—C13	161.39 (9)	C21—C22—C18	108.5 (2)
C15—Fe2—C13	67.98 (7)	C21—C22—Fe2	70.04 (12)
C17—Fe2—C13	40.46 (7)	C18—C22—Fe2	69.82 (12)
C14—Fe2—C13	40.63 (7)	C21—C22—H22	125.7
N1—C1—C5	123.16 (16)	C18—C22—H22	125.7
N1—C1—C2	128.49 (17)	Fe2—C22—H22	125.7
C5—C1—C2	108.21 (17)	O1—C11—O2	124.19 (17)
N1—C1—Fe1	130.32 (13)	O1—C11—N1	125.81 (17)
C5—C1—Fe1	69.15 (10)	O2—C11—N1	109.99 (15)
C2—C1—Fe1	69.40 (10)	O2—C12—H12A	109.5
C3—C2—C1	107.45 (18)	O2—C12—H12B	109.5
C3—C2—Fe1	69.28 (11)	H12A—C12—H12B	109.5
C1—C2—Fe1	70.06 (10)	O2—C12—H12C	109.5
C3—C2—H2A	126.3	H12A—C12—H12C	109.5
C1—C2—H2A	126.3	H12B—C12—H12C	109.5
Fe1—C2—H2A	126.3	O3—C23—O4	124.43 (17)
C4—C3—C2	108.49 (18)	O3—C23—N2	125.99 (17)
C4—C3—Fe1	69.48 (12)	O4—C23—N2	109.58 (15)
C2—C3—Fe1	70.15 (11)	O4—C24—H24A	109.5
C4—C3—H3	125.8	O4—C24—H24B	109.5
C2—C3—H3	125.8	H24A—C24—H24B	109.5
Fe1—C3—H3	125.8	O4—C24—H24C	109.5
C3—C4—C5	108.27 (18)	H24A—C24—H24C	109.5
C3—C4—Fe1	70.04 (11)	H24B—C24—H24C	109.5
C5—C4—Fe1	70.02 (11)	C11—N1—C1	124.97 (15)
C3—C4—H4	125.9	C11—N1—H1	118.5 (14)
C5—C4—H4	125.9	C1—N1—H1	116.5 (14)
Fe1—C4—H4	125.9	C23—N2—C13	125.23 (15)
C1—C5—C4	107.56 (18)	C23—N2—H2	118.8 (15)
C1—C5—Fe1	70.37 (10)	C13—N2—H2	115.9 (15)
C4—C5—Fe1	69.16 (11)	C11—O2—C12	116.10 (16)
C1—C5—H5	126.2	C23—O4—C24	116.42 (17)

### Hydrogen-bond geometry (Å, º)

**Table:**

*D*—H···*A*	*D*—H	H···*A*	*D*···*A*	*D*—H···*A*
N1—H1···O3^i^	0.82 (2)	2.18 (2)	2.971 (2)	162.5 (19)
N2—H2···O1	0.80 (2)	2.18 (2)	2.9605 (19)	166 (2)

Symmetry code: (i) *x*+1, *y*, *z*.
