# Supplementary figures and images for: Crystal structure of methyl N-ferro­cenyl­carbamate
Source: Acta Crystallogr E Crystallogr Commun. 2015 Jan 17;71(Pt 2):m30. doi: 10.1107/S2056989015000043 (PMC4384608; doi:10.1107/S2056989015000043)

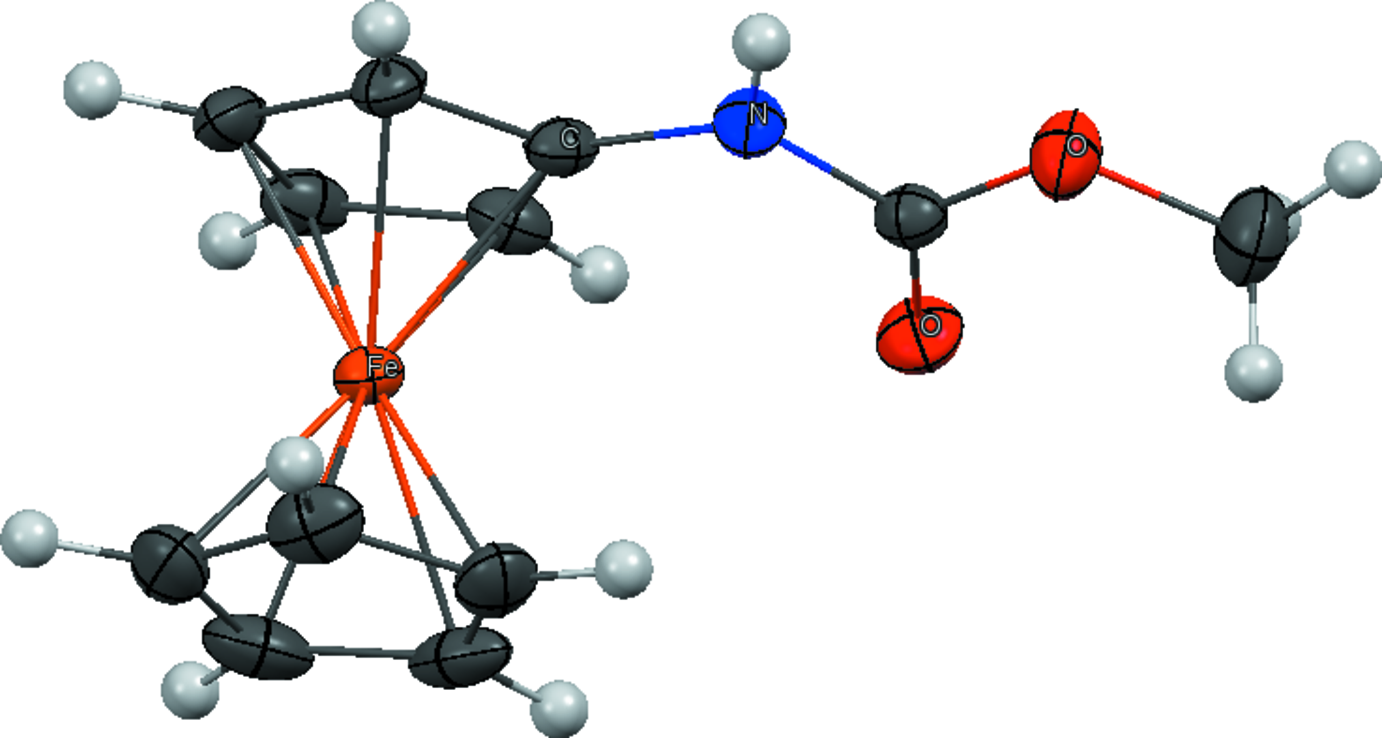

Supplement: Supplementary file 4 [file e-71-00m30-fig1.tif]

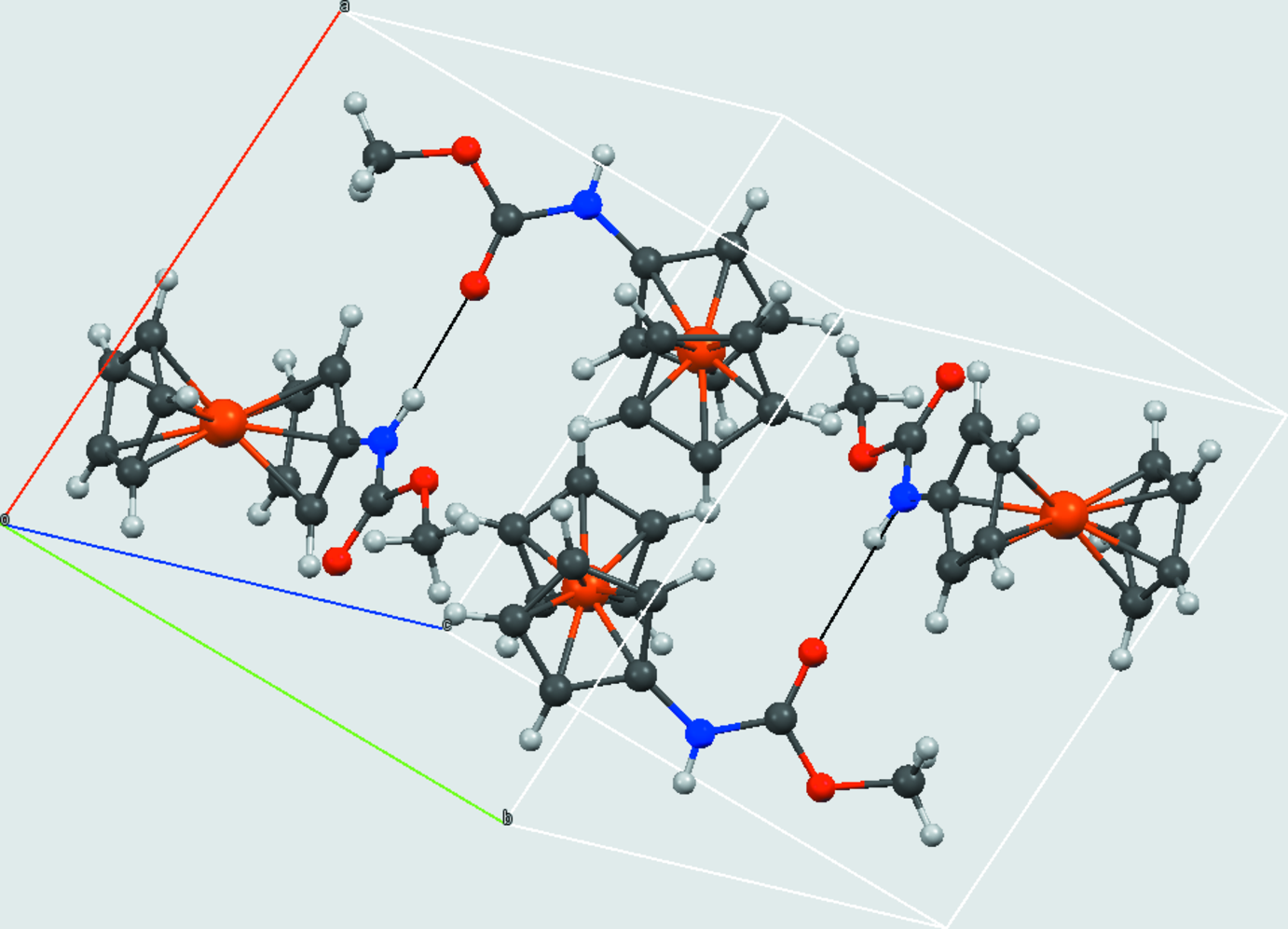

Supplement: Supplementary file 5 [file e-71-00m30-fig2.tif]
